# Inhibition of autophagy as a treatment strategy for p53 wild-type acute myeloid leukemia

**DOI:** 10.1038/cddis.2017.317

**Published:** 2017-07-13

**Authors:** Hendrik Folkerts, Susan Hilgendorf, Albertus T J Wierenga, Jennifer Jaques, André B Mulder, Paul J Coffer, Jan Jacob Schuringa, Edo Vellenga

**Affiliations:** 1 Department of Experimental Hematology, Cancer Research Center Groningen, University Medical Center Groningen, University of Groningen, Groningen, The Netherlands; 2 Department of Laboratory Medicine, University Medical Center Groningen, Groningen, The Netherlands; 3 Regenerative Medicine Center, University Medical Center Utrecht, Utrecht, The Netherlands; 4 Center of Molecular Medicine, University Medical Center Utrecht, Utrecht, The Netherlands

## Abstract

Here we have explored whether inhibition of autophagy can be used as a treatment strategy for acute myeloid leukemia (AML). Steady-state autophagy was measured in leukemic cell lines and primary human CD34^+^ AML cells with a large variability in basal autophagy between AMLs observed. The autophagy flux was higher in AMLs classified as poor risk, which are frequently associated with TP53 mutations (TP53^mut^), compared with favorable- and intermediate-risk AMLs. In addition, the higher flux was associated with a higher expression level of several autophagy genes, but was not affected by alterations in p53 expression by knocking down p53 or overexpression of wild-type p53 or p53^R273H^. AML CD34^+^ cells were more sensitive to the autophagy inhibitor hydroxychloroquine (HCQ) than normal bone marrow CD34^+^ cells. Similar, inhibition of autophagy by knockdown of ATG5 or ATG7 triggered apoptosis, which coincided with increased expression of p53. In contrast to wild-type p53 AML (TP53^wt^), HCQ treatment did not trigger a BAX and PUMA-dependent apoptotic response in AMLs harboring TP53^mut^. To further characterize autophagy in the leukemic stem cell-enriched cell fraction AML CD34^+^ cells were separated into ROS^low^ and ROS^high^ subfractions. The immature AML CD34^+^-enriched ROS^low^ cells maintained higher basal autophagy and showed reduced survival upon HCQ treatment compared with ROS^high^ cells. Finally, knockdown of ATG5 inhibits *in vivo* maintenance of AML CD34^+^ cells in NSG mice. These results indicate that targeting autophagy might provide new therapeutic options for treatment of AML since it affects the immature AML subfraction.

Acute myeloid leukemia (AML) is characterized by the accumulation of immature blast cells in the bone marrow, resulting in a disruption of normal hematopoiesis. The growth advantage of leukemic cells over the normal hematopoietic stem and progenitor cells (HPSC) is linked to a perturbation in differentiation, metabolic and cell survival programming, as a result of a number of genetic and epigenetic defects.^1, 2, 3^ Transcriptome studies have demonstrated that the expression patterns of apoptotic and anti-apoptotic genes are significantly different between AML CD34^+^ cells compared with CD34^+^ cells derived from healthy subjects.^4, 5^

HPSC homeostasis requires macroautophagy (here referred to as autophagy), which is an alternative cell survival program involved in degradation of redundant organelles and proteins.^6, 7, 8^ Autophagic flux in normal HSPC is most prominent in the immature CD34^+^CD38^−^ subfraction and declines in more differentiated myeloid cells.^9^ Maintenance of an adequate level of autophagy is essential for HPSC homeostasis. Previous studies have shown that lentiviral knockdown of the essential autophagy genes ATG5 and ATG7 results in impaired engraftment of cord blood (CB) CD34^+^ cells in NSG mice.^9, 10^ In addition, ATG7^null^ or ATG5^null^ mice develop anemia and during long-term follow-up myelodysplasia.^11, 12, 13^ Recent studies in myeloid leukemia have suggested that in AML the autophagy machinery might be disrupted, resulting in intracellular accumulation of damaged mitochondria and increased levels of reactive oxygen species (ROS), with high ROS levels potentially promoting leukemic transformation.^12, 14, 15^ In contrast, other studies have shown that leukemic cells require functional autophagy during leukemia maintenance.^16, 17, 18^ In addition, autophagy can be an escape mechanism utilized by leukemic cells after treatment with chemotherapeutics such as mTOR- and HDAC inhibitors.^19, 20, 21, 22, 23, 24, 25^ Together, this suggests a greater dependency of AML cells on these effector pathways. The aim of our study was to determine whether inhibiting autophagy can provide an additional means to impair leukemic stem cell (LSC) functionality. We demonstrated that AML CD34^+^ cells are more susceptible for autophagy inhibition than normal CD34^+^ cells. P53 is an important effector pathway in the observed apoptotic responds, triggered by inhibition of autophagy.

## Results

### Leukemic cell lines with an increased autophagic flux are more dependent on autophagy for their survival

**Figure 1:**
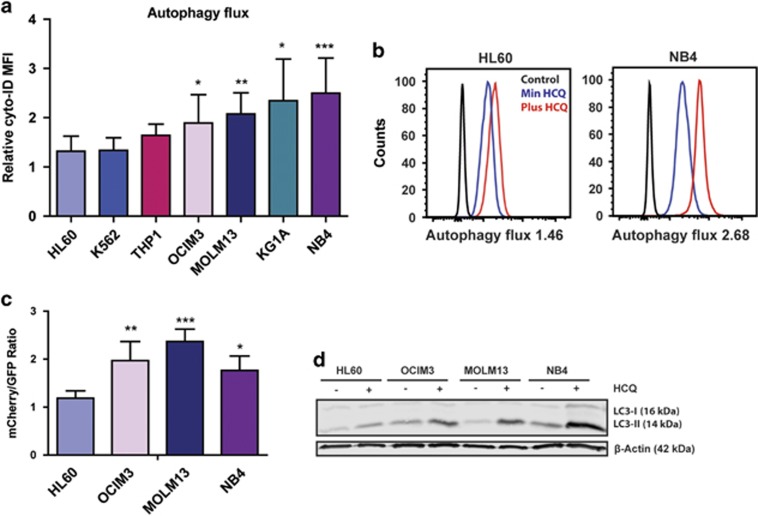
Variation in autophagy flux between different leukemic cell lines. (**a**) Relative accumulation of autophagosomes after overnight treatment with 20 *μ*M HCQ measured by staining with Cyto-ID in a panel of leukemic cell lines (*N*=7). (**b**) Representative FACS plots showing mean fluorescent intensity of Cyto-ID, with or without HCQ treatment. (**c**) mCherry/GFP ratio in a panel of leukemic cell lines transduced with mCherry-GFP-LC3. (**d**) Representative western blot of LC3-II accumulation after HCQ in cell lines, *β*-actin was used as loading control. Error bars represent S.D.; *, ** or *** represents *P*<0.05, *P*<0.01 or *P*<0.001, respectively

During autophagy double-membrane vesicles called autophagosomes are formed, which fuse with lysosomes.^6^ It is important not only to measure the steady-state number of autophagosomes but also the turnover.^26^ This can be done by staining cells with Cyto-ID, a dye that selectively labels autophagic vacuoles. The relative increase in Cyto-ID signal after overnight incubation with hydroxychloroquine (HCQ) is considered to be the autophagy flux.^9^ In the tested cell lines autophagy flux varied, HL60 cells had a significantly lower flux as compared with OCIM3, MOLM13, KG1A and NB4 cells (Figures 1a, b and Supplementary Table S1). These results were confirmed by using alternative methods for analyzing autophagy flux. First, cell lines expressing GFP-ATG8/LC3 were treated with or without the autophagy inhibitor Bafilomycin-1A (BAF). The relative accumulation of GFP-ATG8/LC3 puncta upon BAF treatment is indicative for the level of autophagy flux (Supplementary Figure S1A). Representative pictures of GFP-ATG8/LC3 puncta accumulation in NB4 cells are depicted in Supplementary Figure S1B. In addition, autophagic flux was determined by tandem fluorescent-tagged LC3 reporter (Figure 1c) and relative accumulation of LC3-II by western blotting (Figure 1d, Supplementary Figures S1C and D). To confirm that the observed autophagic flux measurements in combination with HCQ where autophagy specific, HL60 and NB4 cells were pre-treated with 5 mM 3-methyladenine (PI3K inhibitor) or 10 *μ*M SBI-0206965 (ULK1 inhibitor), thereby blocking autophagosome formation. By inhibiting PI3K or ULK1 a near complete block in HCQ-dependent LC3-II accumulation was observed underscoring an autophagy-specific mechanism (Supplementary Figures S1E and F).

**Figure 2:**
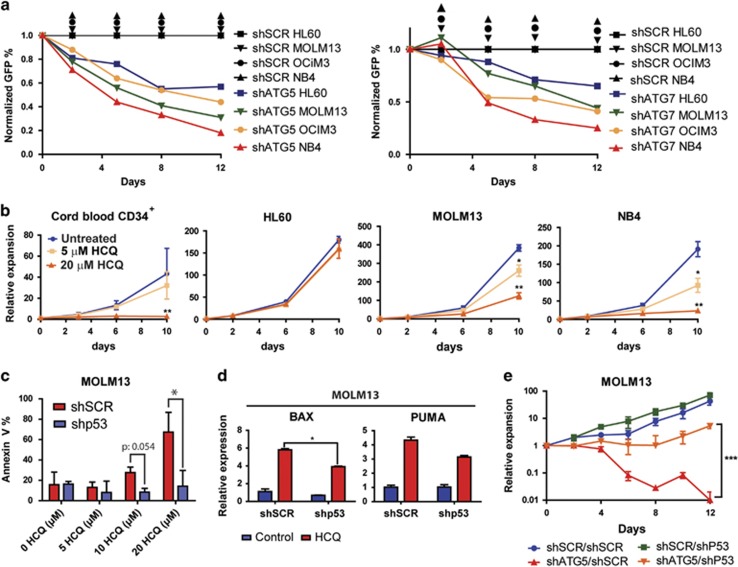
Sensitivity for inhibition of autophagy in leukemic cells. (**a**) Normalized GFP percentages in leukemic cell lines transduced with shSCR-GFP, shATG5-GFP or shATG7-GFP and cultured for 12 days. (**b**) Cumulative growth of leukemia cell lines and cord blood-derived CD34^+^ cells cultured for 10 days in the presence of 0, 5 or 20 *μ*M HCQ. (**c**) Percentage of Annexin-V-positive cells in shSCR or shp53 transduced MOLM13 cells, at day 4 after treatment with different concentrations of HCQ. (**d**) Quantitative RT-PCR for BAX and PUMA in shSCR and shP53 transduced MOLM13 cells, treated with 20 *μ*M HCQ for 4 days. (**e**) Cell expansion in time of MOLM13 cells double transduced with shp53-GFP or shSCR-GFP in combination with shSCR-mCherry or shATG5-mCherry. The transduced cells were cultured for 12 days. Error bars represent S.D.; *, ** or *** represents *P*<0.05, *P*<0.01 or *P*<0.001, respectively

To validate whether the observed autophagic flux was functionally relevant, the HL60, MOLM13, OCIM3 and NB4 cell lines were transduced with lentiviral shRNAs, to knockdown the essential autophagy genes ATG5 (shATG5) or ATG7 (shATG7). Each shRNA was selected from a set of five individual shRNAs, which were extensively tested as described previously.^9^ The knockdown efficiency for shATG5 and shATG7 transduced leukemic cell lines was confirmed by q-PCR (Supplementary Figure S2A). Lentiviral-mediated knockdown of atg5 and atg7 resulted in a reduced accumulation of GFP-ATG8/LC3 puncta after BAF treatment (Supplementary Figure S2B), which coincided with a significant reduction in survival (Figure 2b). To validate these findings in an alternative manner, the cell lines were exposed to different concentrations of HCQ during prolonged culture. Survival and expansion after treatment with HCQ was compared with CB CD34^+^ cells. CB CD34^+^ cells showed no impairment in expansion when treated with 5 *μ*M HCQ, while 20 *μ*M HCQ significantly inhibited their expansion (Figure 2b and Supplementary Figure S2C). The cell lines showed variability in survival after HCQ treatment; notably those most susceptible for HCQ had the highest level of autophagic flux (Figures 1, 2b and Supplementary Figure S2C). The reduced survival and proliferation after inhibition of autophagy was at least in part due to increased apoptosis, as determined by annexin-V staining (Figure 2c, red bars and Supplementary Figure S2D). In MOLM13 and NB4 cells increased apoptosis correlated with increased expression of p53 and its transcriptional target genes BAX, PUMA and PHLDA3 (Supplementary Figure S2E).

To investigate the potential role of p53 in the HCQ-induced cell death, MOLM13 and NB4 were transduced with a lentiviral shRNAs, targeting TP53 (shp53; Supplementary Figure S2A). The p53 status of used leukemic cell lines is indicated in Supplementary Table S1. Compared with shSCR-transduced cells, shp53-transduced cells did not show an apoptotic response to treatment with different HCQ concentrations (Figure 2c, blue bars and Supplementary Figure S2F). Moreover, knockdown of p53 prevented HCQ-dependent expression of pro-apoptotic BAX and PUMA (Figure 2d and Supplementary Figure S2G). In contrast, TP53^null^ HL60 cells, with low basal autophagy (Figure 1a), did not display induction of apoptosis (data not shown) or a strong reduction of expansion upon HCQ treatment (Figure 2b). Finally, p53^wt^ cell lines MOLM13 and OCIM3 were double transduced with shSCR- or shP53-GFP in combination with shSCR- or shATG5-mCherry. As expected, knockdown of ATG5 provided a strong reduction in expansion, which could be rescued by additional knockdown of p53. However, following longer follow-up the rescue by shp53 gradually declined (Figure 2e and Supplementary Figure S2H).

### Variation in autophagy levels between different AMLs independently of the differentiation status

**Figure 3:**
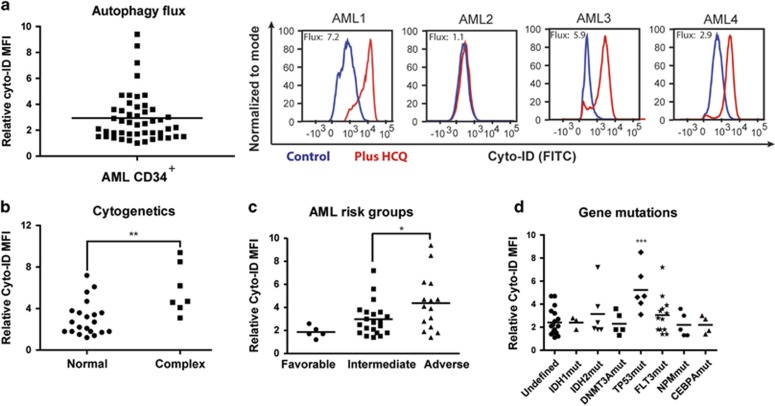
Variation in autophagy levels between different AMLs, independent of the differentiation status. (**a**) Left panel, for autophagic flux measurements (relative Cyto-ID accumulation) in AML CD34^+^ blasts (*n*=51), AML CD34^+^ cells were cultured for 3 days on MS5 stromal layer before overnight incubation with 20 *μ*M HCQ. Right panel, representative FACS plot showing the accumulation of Cyto-ID after treatment with HCQ. (**b**) Autophagy flux in AMLs with normal karyotype *versus* complex cytogenetic abnormalities. (**c**) Autophagy flux in AMLs according to the various ELN risk groups. (**d**) Autophagy flux in AML CD34^+^ cells according to commonly mutated genes in AML. Error bars represent S.D.; *, ** or *** represents *P*<0.05, *P*<0.01 or *P*<0.001, respectively

Next, we analyzed the expression of autophagy genes and the functional consequences in patients AML CD34^+^ cells. In total, 51 AML patients were studied; the clinical characteristics of this cohort are described in Supplementary Tables S2 and S3. For studying a homogeneous AML cell population *in vitro*, the CD34^+^ AML subfraction was sorted and analyzed. Quantitative PCR studies demonstrated that essential autophagy genes ATG5 and ATG7 are more highly expressed in a subset of AMLs compared with CD34^+^ normal bone marrow cells (Supplementary Figure S3A and Supplementary Table S4). In addition, expression levels of autophagy genes in AML and normal bone marrow were assessed in publicly available expression data sets (Bloodspot expression database^27^). Expression of a subgroup of autophagy genes was higher in AML compared with normal HSCs, especially genes involved in the mTOR-dependent ULK1 complex or LC3 lipidation (Supplementary Figure S3B).^27^ To investigate the functional consequences of this observation, we measured autophagy flux in AML CD34^+^ cells (*n*=51). A large variability in autophagic flux was observed, comparable to the results in cell lines (Figures 1, Figures 3a and Supplementary Figure S3C). No difference in autophagic flux was observed between the AML CD34^+^CD38^−^ fraction compared with more mature CD34^+^CD38^+^ fraction (*n*=8; Supplementary Figure S3D). Also, no difference was observed between bone marrow and peripheral blood-derived AML cells (Supplementary Figure S3E). Since AML is clinically a heterogeneous disease, autophagic flux was correlated to a number of clinical relevant parameters, including the French–American–British classification (FAB), cytogenetics, molecular markers and prognostic risk classification. No significant difference in autophagy flux was shown between AML cells belonging to the myeloid (M1–M2) or monocytic lineages (M4–M5) (Supplementary Figure S3F). Cytogenetic analysis revealed that AML patients with complex cytogenetic abnormalities had the highest level of autophagy (Figure 3b). In line with these results, expression of many core autophagy genes was higher in AMLs with complex karyotype compared with other AML subgroups (Supplementary Figure S3B).^27^ When patients were categorized according to ELN criteria^28^ in favorable, intermediate-I, -II and adverse-risk groups, AML CD34^+^ cells belonging to adverse-risk group had significantly higher levels of autophagy compared with the intermediate- or favorable-risk AMLs (Figure 3c). AMLs with mutations in TP53, which were all classified as adverse-risk, had higher autophagic flux (Figure 3d). In contrast, no differences in autophagy levels were observed in AMLs harboring mutations in FLT3, NPM1, IDH1/2, DNMT3A or CEPBA genes (Figure 3d).

**Figure 4:**
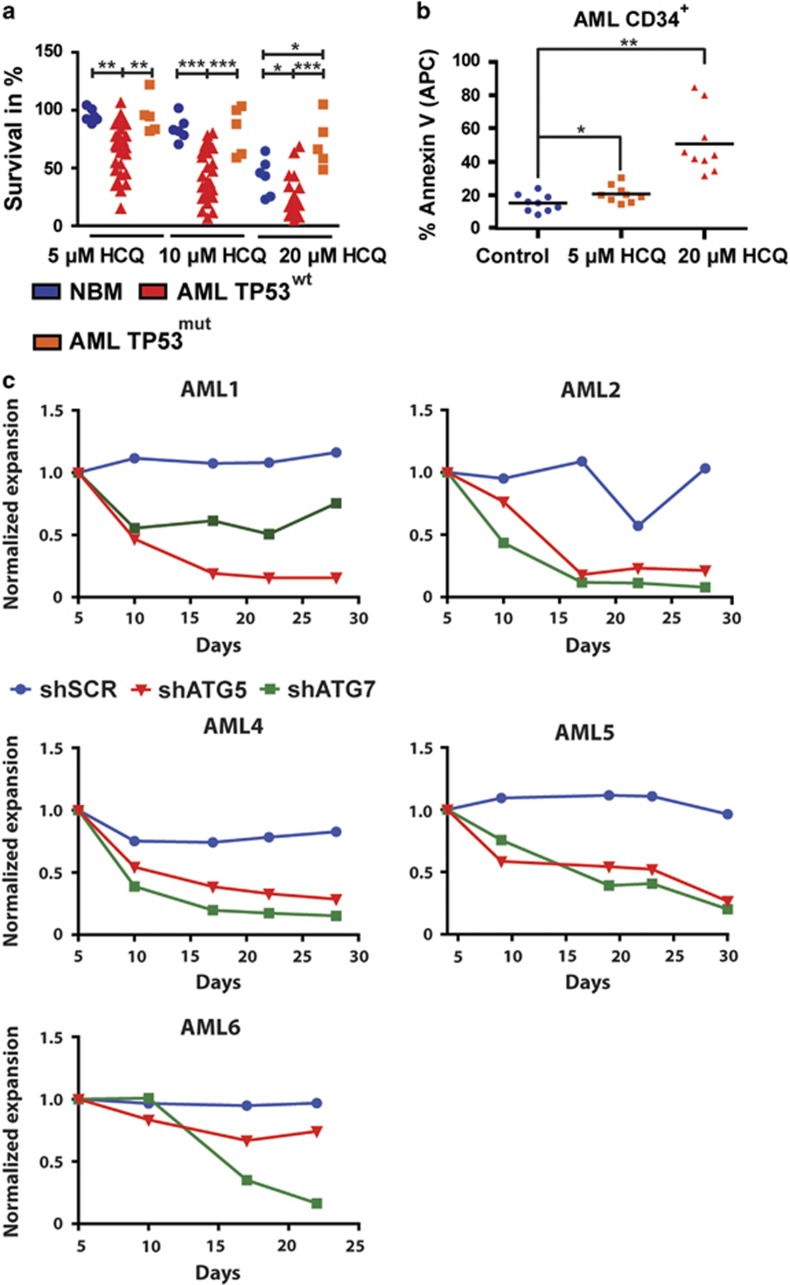
Inhibition of autophagy triggers apoptosis in primary AML CD34^+^ cells. (**a**) Survival of normal bone marrow (NBM) CD34^+^, TP53^wt^ AML CD34^+^ or TP53^mut^ AML CD34^+^ cells were cultured for 3 days on an MS5 stromal layer before treated with 5, 10 or 20 *μ*M HCQ for 48 h. (**b**) Quantification of Annexin-V percentages in AML (*n*=9) after treatment with 5 or 20 *μ*M HCQ. (**c**) Normalized expansion of AML CD34^+^ cells transduced with shSCR, shATG5 or shATG7, cultured on an MS5 stromal layer. Error bars represent S.D.; *, ** or *** represents *P*<0.05, *P*<0.01 or *P*<0.001, respectively

To study the functional relevance of the autophagic flux for survival, AML CD34^+^ cells were treated with 0, 5, 10 or 20 *μ*M HCQ for 72 h. The survival of AML cells was measured over time and compared with normal bone marrow CD34^+^ cells treated in a similar manner. As shown in Figure 4a, a significant dose-dependent increase in sensitivity to HCQ was observed in AML CD34^+^ compared with CD34^+^ cells isolated from healthy controls (20 *μ*M HCQ, 23.0±3.1% *versus* 42.5±6.6% surviving cells, respectively, *P*<0.05). Similarly, inhibition of autophagy in AML CD34^+^ cells resulted in a dose-dependent increase in apoptosis as measured by annexin-V positivity (Figure 4b and Supplementary Figure S4A). In contrast to observations in leukemic cell lines, no correlation was observed between the level of autophagic flux and the sensitivity for HCQ. To validate the dependency on autophagy in an alternative manner, AML CD34^+^ (*n*=5) were transduced with either shATG5 or shATG7, and expansion on an MS5 stromal layer was measured over time. A strong decrease in cell expansion was observed in response to ATG5 or ATG7 downregulation in comparison with shSCR-transduced AML cells (Figure 4c, Supplementary Figures S4B and C).

### Inhibition of autophagy triggers a p53-dependent increase in apoptosis in AML CD34^+^ cells

**Figure 5:**
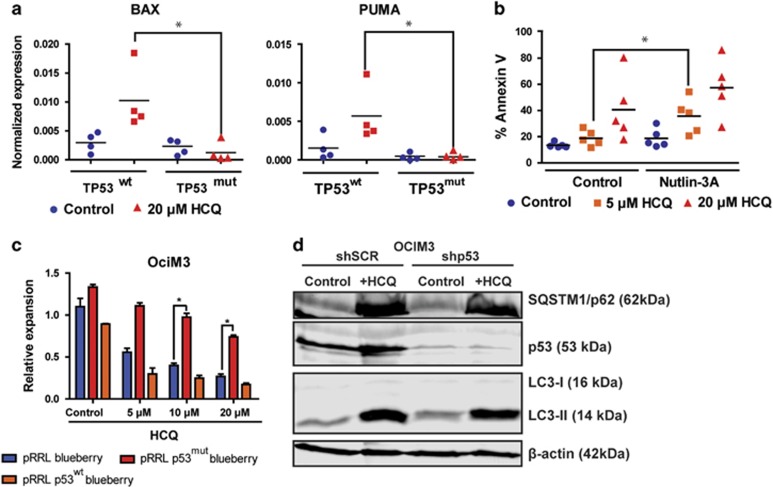
TP53 mutant AMLs are resistant for HCQ-induced apoptosis. (**a**) Gene expression of BAX and PUMA determined by quantative RT-PCR in TP53^wt^ (*n*=4) or TP53^mut^ (*n*=4) AMLs. AML CD34^+^ cells were cultured for 3 days on an MS5 stromal layer before 72 h incubation with 20 *μ*M HCQ. (**b**) Percentage of Annexin-V-positive cells in TP53^wt^ AML CD34^+^ cells treated with 5 or 20 *μ*M HCQ in conjunction with or without Nutlin-3A. (**c**) Cell counts of OCIM3 cells transduced with pRRL-mBlueberry, pRRL-P53mut-mBlueberry or pRRL-P53wt-mBlueberry, treated with different concentrations of HCQ. (**d**) Western blot showing LC3-II, sqstm1/p62 and p53 protein expression in OCIM3 cells transduced with shSCR or shP53 treated overnight with or without 20 *μ*M HCQ. Error bars represent S.D.; * represents *P*<0.05

Since we observed that some AML CD34^+^ samples were less sensitive for HCQ, we compared the sensitivity of wild-type TP53 (TP53^wt^) to those harboring TP53 mutations (TP53^mut^). As shown in Figure 4a, TP53^mut^ AML CD34^+^ cells (*n*=5, Supplementary Table S3) were significantly less sensitive at 5, 10 or 20 *μ*M HCQ compared with TP53^wt^ cells (20 *μ*M HCQ, 71.8.±9.8% *versus* 23.0±3.1% surviving cells, respectively, *P*<0.0001). To characterize further differences in responsiveness between TP53^wt^ and TP53^mut^ patient-derived cells, AML CD34^+^ TP53^wt^ cells (*n*=5) or TP53^mut^ cells (*n*=4) were treated with HCQ, and p53-dependent transcriptional target gene expression patterns were analyzed. In patients with TP53 mutations both homozygous and heterozygous TP53 mutations were observed. Basal levels of BAX, PUMA and p21 mRNA expression were lower in TP53^mut^ cells compared with TP53^wt^ AML CD34^+^ cells. Interestingly, in contrast to TP53^wt^ cells, expression levels of pro-apoptotic BAX and PUMA were not increased upon HCQ treatment in TP53^mut^ AML CD34^+^ cells, suggesting that the apoptotic response was severely dampened in these cells (Figure 5a). To confirm the role of p53 in the HCQ-mediated effects, TP53^wt^ AML cells were co-treated with Nutlin-3A, which stabilizes p53 by inhibition of MDM2. The combined used of HCQ and Nutlin-3A significantly enhanced the apoptotic effect compared with HCQ alone in TP53^wt^ AML CD34^+^ cells (Figure 5b). To verify these findings in an alternative manner p53^wt^ and mutant TP53^R273H^ were overexpressed in p53^wt^ OCIM3 leukemic cells and subsequently treated them with increasing concentrations of HCQ. TP53^R273H^ is described as gain-of-function mutation associated with drug resistance. Overexpression of p53^wt^ enhanced the HCQ-dependent apoptotic response and resulted in reduced survival compared with control (Supplementary Figure S5A). In contrast, overexpression of mutant TP53^R273H^ rendered the AML cells more resistant to HCQ treatment (Figure 5c and Supplementary Figure S5A). However, overexpression of p53^wt^ or TP53^R273H^ in OCIM3 cells did not affect the autophagic flux as determined by Cyto-ID (Supplementary Figure S5B). Comparable results were obtained in the context of p53 knockdown in OCIM3 and MOLM13 cells. No change in accumulation of LC3-II or sqstm1/p62 was observed. (Figure 5d and Supplementary Figure S5C). Also in normal CB CD34^+^ cells overexpression of p53^wt^ or TP53^R273H^ did not affect the levels of autophagy (relative Cyto-ID values; control 2.3±0.4-fold, p53^wt^ 2.2±0.6-fold or p53^mut^ 2.1±0.3-fold). Together, these results indicate that inhibition of autophagy initially triggers a p53-dependent apoptotic response, which is severely dampened in AML CD34^+^ cells harboring mutations in the TP53 gene irrespective of the autophagy flux.

### AML CD34^+^ROS^low^ cells have a higher autophagic flux

**Figure 6:**
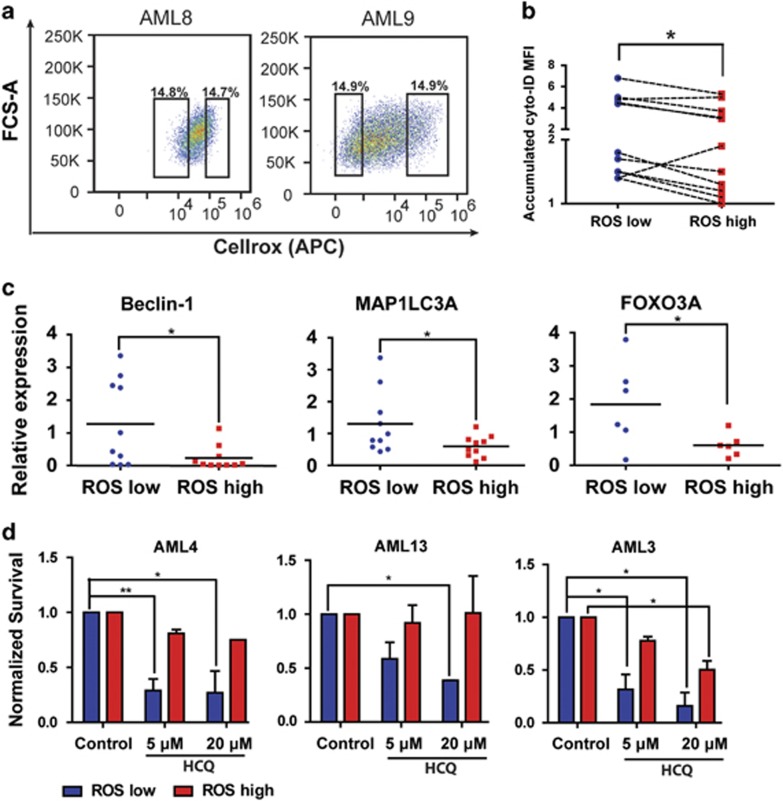
Autophagy is higher in the ROS^low^ population of AML blasts. (**a**) Representative FACS plots showing CellROX staining in freshly isolated AML CD34^+^ cells. (**b**) Relative Cyto-ID levels in ROS^high^ and ROS^low^ fractions of AML CD34^+^ cells (*n*=11). (**c**) Gene expression of Beclin-1 and MAP1LC3A in freshly sorted AML CD34^+^ROS^high^ and CD34^+^ROS^low^ cells. (**d**) Survival of FACS-sorted ROS^high^ and ROS^low^ AML CD34^+^ cells, cultured for 3 days on an MS5 stromal layer before treated for 48 h with different concentrations HCQ. Error bars represent S.D., * or ** represents *P*<0.05 or *P*<0.01 respectively

We did not observe differences in autophagy in more immature CD34^+^CD38^−^ *versus* more mature CD34^+^CD38^+^ blast (Supplementary Figure S3D). To determine whether there is still variability in the level of autophagy within the AML CD34^+^ fraction, we separated the AML CD34^+^ subfraction into ROS^low^ and ROS^high^ cells. A recent study has shown that ROS^low^ AML cells are enriched for LSCs by using *in vitro* as well as *in vivo* assays.^29^ We identified the ROS^low^ and ROS^high^ AML CD34^+^ by sorting the 15% low and high subfractions based on the CellROX mean fluorescent intensity (MFI) in the AML CD34^+^ cell population (Figure 6a). A significant distinction in CellROX MFI was demonstrated in AML CD34^+^ (*n*=14) ROS^high^ compared with ROS^low^ cells (Figure 6a and Supplementary Figure S6A). Sorted ROS^low^ cells exhibited more immature morphology, as determined by the relative size of the nucleus to the cytoplasm. Representative pictures of AML cells from sorted ROS^low^ and ROS^high^ AMLs are shown in Supplementary Figure S6B. Interestingly, ROS^low^ cells maintained a significantly higher autophagic flux compared with the ROS^high^ AML CD34^+^ cells, within the same patient sample, as determined by Cyto-ID (Figures 6b, *P*<0.01 and Supplementary Figure S6C). In addition, sorted ROS^low^ and ROS^high^ subfractions AML CD34^+^ cells were treated overnight with HCQ and subsequently accumulation LC3-II was detected by western blotting. A higher accumulation was shown in the ROS^low^ AML cells (Supplementary Figure S6D). qRT-PCR analysis demonstrated a significantly higher expression of BCL-2 in the ROS^low^ AML CD34^+^ cells (Supplementary Figure S6E). In addition, higher expression of the autophagy genes Beclin-1 and MAP1LC3A and the autophagy regulator FOXO3A was observed in ROS^low^ AML CD34^+^ cells compared with the ROS^high^ CD34^+^ cells (Figure 6c).^30, 31^ In contrast, expression of other key autophagy genes and major ROS scavengers such as SOD1, SOD2 and Catalase was comparable between both fractions (data not shown).

To evaluate growth characteristics and the functional relevance of autophagy in the distinct AML CD34^+^ subpopulations (*n*=4), FACS-sorted AML CD34^+^ ROS^low^ and ROS^high^ cells were cultured on MS5 bone marrow stromal cells. The ROS^low^ AML CD34^+^ cells exhibited long-term expansion in comparison with the ROS^high^ CD34^+^ cells (week 5; ROS^low^ 7.1-fold±2.1 *versus* ROS^high^ 1.6-fold±0.4 (*n*=6, *P*≤0.05)). Next, ROS^low^ and ROS^high^ fractions were treated with 5 or 20 *μ*M HCQ for 48 h and survival was determined (Figure 6d). ROS^low^ cells were more sensitive to HCQ treatment compared with ROS^high^ cells, correlating with increased apoptosis (Supplementary Figure S6F). Since mitochondria have an important role in ROS production, we evaluated mitochondrial mass in AML CD34^+^ cells in both the ROS^low^ and ROS^high^ subfractions. AML CD34^+^ ROS^low^ cells had a lower mitochondrial mass compared with ROS^high^ AML CD34^+^ cells (*n*=11, *P*<0.0001; Supplementary Figure S6G).

### Knockdown of ATG5 inhibits myeloid leukemia maintenance *in vivo*

**Figure 7:**
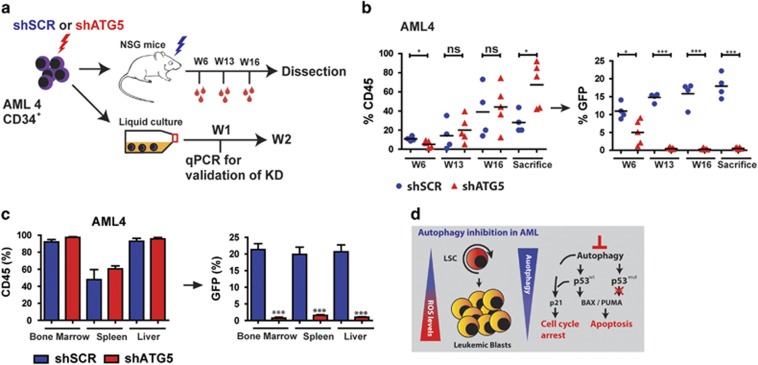
Knockdown of ATG5 in AML CD34^+^ blasts results in impaired engraftment. (**a**) Experimental set-up. (**b**) Left panel: engraftment levels measured by huCD45%. Right panel: the GFP% within huCD45^+^ population. Each dot represents data from a single mouse, shSCR (*N*=4) and shATG5 (*N*=5). (**c**) Engraftment (percentage huCD45) at time of killing in bone marrow, spleen and liver and the GFP% within the huCD45^+^ population. (**d**) Summarizing Model: LSCs are enriched in the ROS^low^ fraction of AML blasts. ROS^low^ cells maintain a higher basal autophagy flux and have a lower mitochondrial mass compared with ROS^high^ cells. Right part: short-term genetic or pharmaceutical Inhibition of autophagy triggered a p53-dependent apoptotic response in p53 wild-type AMLs, which was severely dampened in p53 mutant AMLs. Error bars represent S.D.; * or ***represents *P*<0.05 or *P*<0.001, respectively

Based on the observations that ATG5 and ATG7 knockdown reduce the expansion of AML CD34^+^ cells *in vitro*, we determined whether this would also occur *in vivo.* To exclude the possibility that the knockdown of ATG5 or ATG7 affected cell migration, *in vitro* transwell experiments were performed with the OCIM3 and MLOM13 cell line. In both cell lines, the SDF1-mediated migration was not affected by the knockdown of ATG5 or ATG7 (Supplementary Figure S7D). Subsequently AML CD34^+^ cells were transduced with the shATG5 or shSCR-GFP and transplanted in immunodeficient NSG mice, as outlined in Figure 7a. Transplanted AML blasts were at least 14% GFP positive at the time of injection (Supplementary Figure S7A) and ATG5 knockdown was confirmed by qRT-PCR (Supplementary Figures S7B and C). The time for the onset of leukemia was determined by measuring the percentage of huCD45 in peripheral blood. While GFP levels for shSCR remained stable at around ~15%, the contribution of the shATG5-transduced cells to the engrafted AML cells was significantly reduced, starting from week 6 (Figure 7b and Supplementary Figure S7E). After killing, we observed high engraftment levels in bone marrow, spleen and liver as determined by the percentage of CD45. The contribution of shSCR-GFP-transduced cells within the CD45 compartment was stable around ~20% in all studied organs. On the contrary, the percentage of shATG5-GFP-transduced cells within the CD45 compartment was strongly decreased (Figure 7c). The engrafted human AML cells were all of myeloid origin, as determined by CD33 expression (Supplementary Figure S7F). These results demonstrate that autophagy is also essential for leukemia maintenance *in vivo*.

## Discussion

The aim of our study was to determine whether inhibiting autophagy can provide an alternative means to impair LSC functionality. AML CD34^+^ cells were susceptible for autophagy inhibition, which was demonstrated by *in vitro* and *in vivo* experiments. *In vitro* studies indicated that the subfraction of ROS^low^ AML CD34^+^ cells had the highest autophagic flux and were more susceptible to HCQ treatment when compared with ROS^high^ AML CD34^+^ cells. The AML ROS^low^ subfraction is further characterized by lower mitochondrial mass and elevated BCL-2, FOXO3A and Beclin-1 expression. These results are of interest since a previous study has shown that ROS^low^ AML CD34^+^ cells are enriched for LSCs.^29^ Similarly, murine ROS^low^ HSPC are enriched for stem cells.^32^ In the studied AMLs, the autophagy flux was most pronounced in adverse-risk group with complex cytogenetic abnormalities thath are frequently associated with TP53 mutations. Transcriptome data revealed a significant higher expression of autophagy genes in the AML subgroup with complex karyotype. It has been suggested that the adverse-risk AMLs have a higher number of LSCs compared with favorable-risk AMLs, which might have consequences for the measured level of autophagy.^33, 34^ Although the high autophagy flux was connected with complex karyotype and TP53 mutations, modulation of p53 in normal or leukemic cells by p53 knockdown or ectopic overexpressing p53^mut^ did not affect the autophagy flux. Therefore, the high autophagic flux in the AML CD34^+^ subfraction might be an intrinsic property as consequences of an adaptive response to constitutive metabolic stress linked to the (epi)genetic mutations.

Inhibition of autophagy in leukemic cells might limit nutrient availability in cells, causing metabolic stress and consequently apoptosis. Moreover, impaired autophagy in hematopoietic cells has been associated with increased mitochondrial mass, resulting in ROS accumulation.^8, 9, 15^ In turn, excessive ROS has been shown to cause oxidative DNA damage and consequently premature senescence and HSC exhaustion.^35, 36^ Our study indicates that the p53 pathway, irrespective of the level of autophagy, is an important effector pathway for cell death induced by autophagy inhibition, which has consequences for AMLs with TP53 mutations. TP53^mut^ AML cells show decreased sensitivity for short-term treatment with HCQ and an impaired upregulation of the apoptotic genes PUMA and BAX, indicating that the initial apoptotic response in these cells is strongly impaired.

In view of these findings co-treatment with autophagy inhibitors might only be a promising approach for the treatment of TP53^wt^ AMLs. Similar observations have been made in chronic myeloid leukemia (CML).^37, 38^ The combination of tyrosine kinase inhibitors in combination with autophagy inhibitors resulted in more effective elimination of CML stem cells.^39^ This approach might also be attainable *in vivo* since various studies in patients with solid tumors have shown that high-dose HCQ can block autophagy *in vivo.*^17, 23, 40, 41^ Currently, a second generation of HCQ-derived autophagy inhibitors are being developed, which are more potent in inhibition of autophagy,^42, 43^ thereby increasing the clinical applicability of autophagy inhibition.

In the present study we focused mainly on the role of autophagy during leukemia maintenance. This might be distinct from the role of autophagy during leukemia initiation, as consequences of the emergence of (epi)genetic mutations.^2, 3^ Model systems for leukemia and solid tumors have shown that during malignant transformation, autophagy might be reduced as a result of mutagenesis, resulting in accumulation of mitochondria, ROS-mediated DNA damage and activation of NF-*κ*B signaling.^12, 13^ Likewise, U2AF35 mutations in myelodysplastic syndrome cause abnormal processing of ATG7 pre-mRNA and consequently reduced expression of ATG7.^44^ In addition, a recent study reported mutations of autophagy genes in a small fraction of MDS patients, which might be contributive to malignant transformation.^45^

In summary, our results demonstrate that autophagy has a critical function for AML maintenance and that inhibition of autophagy might be a promising therapeutic strategy in a subgroup of AML patients (summarizing model, Figure 7d).

## Material and methods

### Isolation and culture of human CD34^+^ cells

We obtained umbilical cord blood (UCB) from full-term healthy neonates who were born at the Obstetrics departments of the Martini Hospital and the University Medical Center Groningen (Groningen, the Netherlands). Informed consent was obtained to use UCBs and patients' AML blasts derived from peripheral blood cells or bone marrow in accordance with the Declaration of Helsinki; the protocols were approved by the Medical Ethics Committee of the University Medical Center Groningen (UMCG). Mononuclear cells were isolated from UCB, or peripheral blood or bone marrow from AML patients by Ficol density centrifugation, and CD34^+^ cells were subsequently isolated with the autoMACS pro-separator (Miltenyi Biotec, Amsterdam, the Netherlands).

### Cell culture

Primary AML, normal bone marrow or CB-derived CD34^+^ cells were cultured in suspension or in T25 flasks pre-coated with MS5 stromal cells in Gartners medium: Alpha-MEM (Lonza, Leusden, the Netherlands) supplemented with 12.5% FCS and 12.5% horse serum (Sigma-Aldrich, Saint Louis, MO, USA), 1% penicillin/streptomycin (PAA Laboratories, Dartmouth, MA, USA), 1 *μ*M hydrocortisone (Sigma-Aldrich), 57.2 mM *β*-mercaptoethanol and cytokines: G-CSF, Human TPO agonist; Romiplostim (Amgen, Breda, the Netherlands) and IL-3 (20 ng/ml each).^46^ For the autophagic flux AML CD34^+^ cells were cultured for 3 days on an MS5 stromal layer. Subsequently, the autophagic flux was determined with cyto-ID. The relative increase in Cyto-ID signal after overnight incubation with 20 *μ*M HCQ is considered to be the autophagy flux.^9^ The used concentration and incubation time of HCQ for measuring autophagic flux was validated and is based on maximal accumulation of autophagosomes, without affecting cell viability after overnight incubation with HCQ. AMLs that did not expand were excluded from analysis. The leukemic cell lines HL60, K562, THP1, OCIM3, MOLM13 and NB4 cells were cultured in RPMI 1640, supplemented with 10% FCS and 1% penicillin/streptomycin. KG1A cells were cultured in IMDM (Lonza, Leusden, the Netherlands) 20% FCS and 1% penicillin/streptomycin.

### Flowcytometry analysis

After isolation, cells were resuspended in PBS and subsequently incubated for 30 min at 4 °C with anti-human CD19, CD34, CD38, CD33 and CD45. After incubation, cells were washed and optionally incubated for 30 min at 37 °C using Cyto-ID Autophagy Detection dye (ENZ-51031-0050; Enzo Life Sciences, Raamsdonksveer, The Netherlands). The cells were subsequently washed and analyzed by flow cytometric analysis (FACS). (Additional information can be found in Supplementary Table S5.) All data were analyzed using FlowJo (Tree Star, Ashland, OR, USA) software.

### Apoptosis, ROS and mitochondrial mass measurements

Apoptosis was quantified by staining with APC-conjugated Annexin-V (Beckton Dickinson, Franklin Lakes, NJ, USA) according to the manufacturer’s protocol. ROS analyses were performed by means of CellROX deep red (APC) or CellROX green (FITC; Life Technologies, Landsmeer, the Netherlands), according to the manufacturer’s protocol. Mitochondrial mass was determined with Mitotracker staining (Life Technologies), according to the manufacturer’s protocol. Apoptosis, CellROX and mitochondrial mass were analyzed by FACS.

### Virus production and transduction of CD34^+^ leukemic cells

shATG7 (TRCN0000007586; Sigma-Aldrich) and shATG5 (TRCN0000151474; Sigma-Aldrich) and shP53 vectors were cloned and extensively validated, as previously described.^9^ An shRNA sequence that does not target human genes (referred to as scrambled) was used as a control. TP53^R273H^ or TP53^wt^ were generated by PCR amplification from cDNA obtained from MDA-MB-468 or MOLM13 cells, respectively. Amplified cDNA was subsequently cloned into pRRL-IRES-mBlueberry vector,^47^ using *Eco*R1 restriction sites. Lentiviral virions were produced by transient transfection of HEK 293 T cells with pCMV and VSV-G packing system using Polyethylenimine (Polyscience Inc. Eppelheim, Germany) or FuGENE (Promega, Leiden, the Netherlands). Retroviral virions containing pBABE-puro-mCherry-EGFP-LC3B (kind gift from Prof. Andrew Thorburn, Department of Pharmacology, University of Colorado Cancer Center) were produced by transient transfection of HEK 293 T cells with VSV-G, pAmpho packing system and FuGENE. Viral supernatants were collected and filtered through a 0.2-*μ*m filter and subsequently concentrated using Centriprep Ultracel YM-50 centrifugal filters (Millipore, Billerica, MA, USA). 0.5 × 10^6^ CD34^+^ cells were seeded in Gartners medium supplemented with cytokines (specified previously). Transduction was performed by adding 0.5 ml of ~10 times concentrated viral supernatant to 0.5 ml of medium in the presence of 4 *μ*g/ml polybrene (Sigma-Aldrich). For retroviral transfections, cells were transfected in retronectin-coated 24-well plates.

### Quantitative real-time PCR

Quantitative RT-PCR was used to analyze the mRNA levels of ATG5, Beclin-1, ATG8/LC3, VMP1, ATG10, ATG7, BAX, PUMA, BCL-2 PHLDA3, p21, p53, FOXO3A, SOD1, SOD2 and Catalase. Total RNA was isolated from at least 1 × 10^5^ cells using the RNeasy kit (Qiagen, Venlo, the Netherlands). RNA was reverse transcribed with iScript reverse Transcription kit (Bio-Rad, Veenendaal, the Netherlands). The cDNA obtained was real-time amplified, in iQ SYBR Green Supermix (Bio-Rad), with the CFX connect Thermocycler (Bio-Rad). RPL27 and RPS11 were used as housekeeping genes. The primer sequences are listed in Supplementary Table S6.

### *In vivo* transplantation of AML CD34^+^ cells into NSG mice

For transplantation, 12- to 13-week-old female NSG (NOD.Cg-Prkdcscid IL2rgtm1Wjl/SzJ) mice were purchased from the Central Animal Facility breeding facility at the UMCG. Mouse experiments were performed in accordance with national and institutional guidelines, and all experiments were approved by the Institutional Animal Care and Use Committee of the University of Groningen (IACUC-RuG). General aspects of these experiments have been described previously,^9, 48^ and the detailed experimental approach is described in the Supplementary Methods.

### Statistical analysis

An unpaired two-sided Student’s *t*-test was used to calculate statistical differences. A *P*-value of <0.05 was considered statistically significant.

Additional Materials and Methods can be found in the Supplementary Materials and Supplementary Methods.
