# Supplementary material for: Inhibition of autophagy as a treatment strategy for p53 wild-type acute myeloid leukemia
Source: Cell Death Dis. 2017 Jul 13;8(7):e2927–. doi: 10.1038/cddis.2017.317 (PMC5550863; doi:10.1038/cddis.2017.317)
Supplement: Supplementary Information [file cddis2017317x1.pdf]

Supplementary Figure 1.

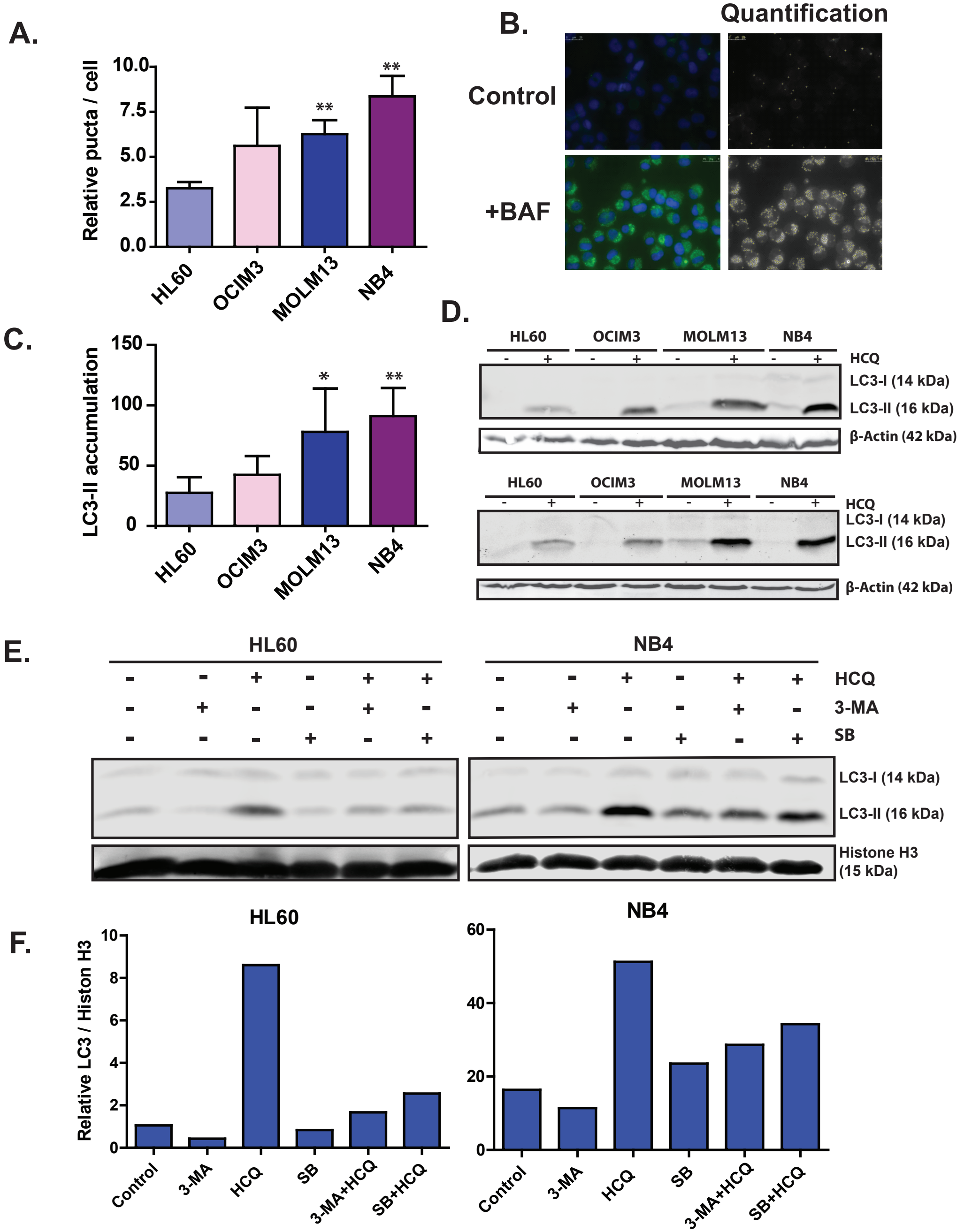

Supplementary Figure 2.

A.

	Knockdown efficiency for shATG5, shATG7 and shP53			
	shSCR	shATG5 (ATG5)	shATG7 (ATG7)	shP53 (p53)
HL60	1,0	0,22 **	0,54 *	na
MOLM13	1,0	0,42 *	0,44 *	0,03 **
OCIM3	1,0	0,25 **	0,63	na
NB4	1,0	0,11 ***	0,41 *	0,15 *

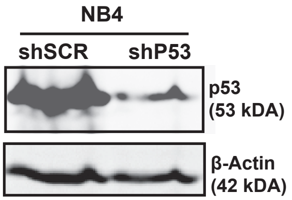

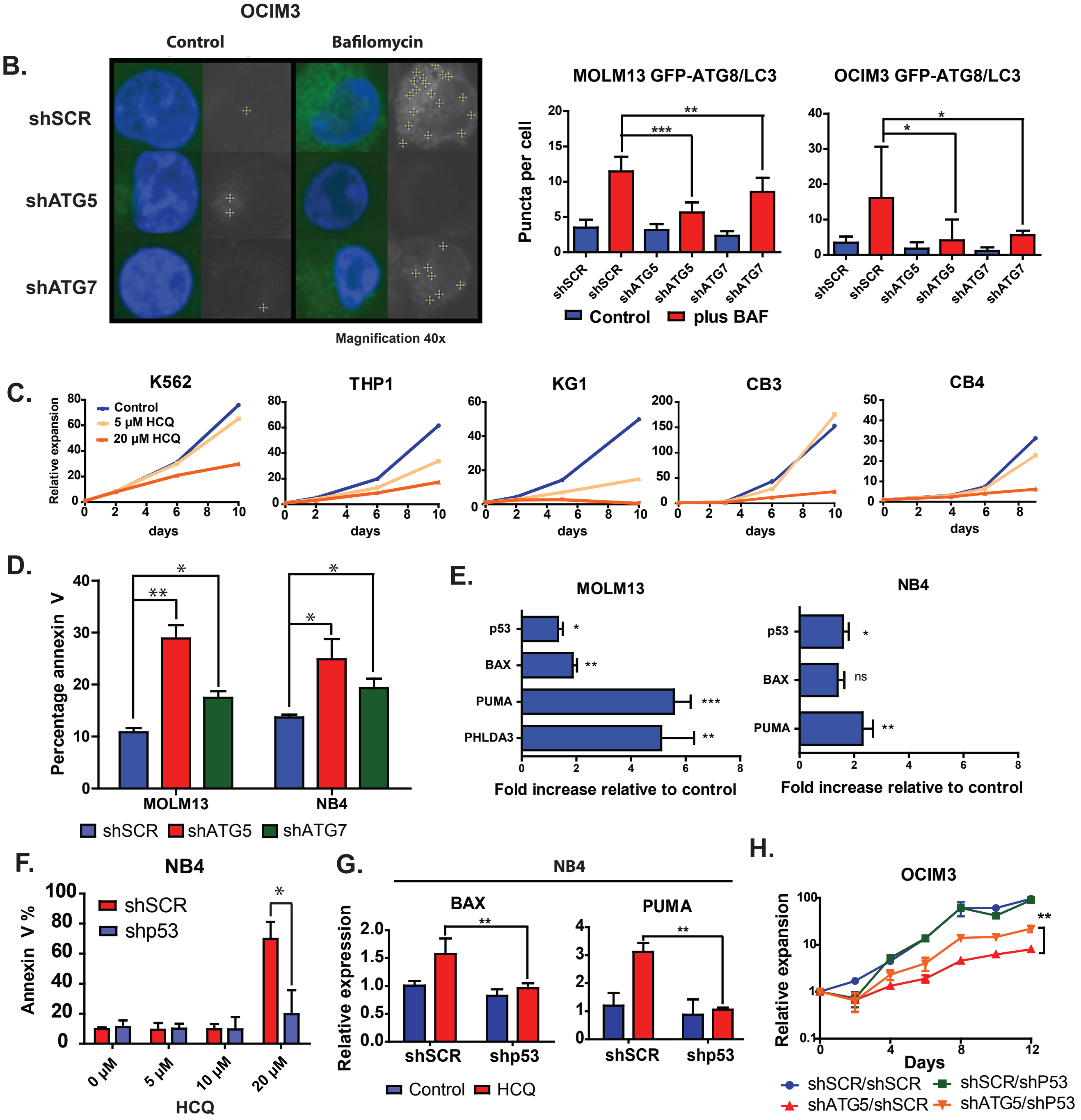

Supplementary Figure 3.

A.

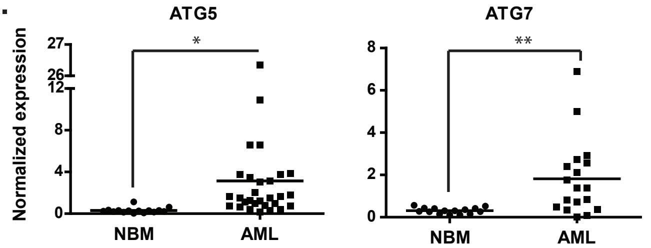

B.

	LC3 Lipidation						mTOR dependent Ulk1 complex			ATG12 complex				
	ATG7	ATG3	MAP1LC3B	ATG4A	ATG4B	ATG4D	ULK1	ATG13	FIP200 / RBCC1	ATG7	ATG5	ATG12	ATG16L1	ATG16L2
AML t(15;17)	1,21	1,72	1,74 *	1,39 *	1,44 **	1,41 *	2,22 **	3,20 ***	1,02	1,21	1,57	2,20 ***	0,77	1,25
AML inv(16)/t(16;16)	1,55	2,35 ***	2,22 ***	1,11	1,39 *	1,31	2,33 ***	1,77 ***	0,91	1,55	1,39	1,89 ***	0,77	0,98
AML t(8;21)	1,68	2,11 **	1,87 *	1,07	1,45 **	1,20	1,40	2,06 ***	1,17	1,68	1,54	2,30 ***	0,84	1,29
AML t(11q23)/MLL	1,71 *	3,07 ***	2,00 **	1,35 **	1,65 ***	1,95 ***	1,93 *	2,07 ***	0,78	1,71 *	1,77 **	2,46 ***	0,81	1,13
AML complex	0,98	3,36 ***	4,08 ***	1,51 **	2,00 ***	3,07 ***	2,97 ***	2,36 ***	1,64	0,98	2,06 ***	1,93 ***	1,11	0,49
HSCs	1,00	1,00	1,00	1,00	1,00	1,00	1,00	1,00	1,00	1,00	1,00	1,00	1,00	1,00

	mTOR independent regulation					Class III Pi3K dependent regulation			p62
	BIF-1 / SH3GLB1	ATG14L	UVRAG	AMBRA1	RAB5	Beclin1 (BECN1)	hVps34	hVps15 / PIK3R4	P62 / SQSTM1
AML t(15;17)	1,86 *	1,03	0,77	1,18	0,99	1,09	0,71	0,85	1,22
AML inv(16)/t(16;16)	2,17 ***	0,91	1,14	1,08	0,90	0,98	0,50 ***	0,69	1,21
AML t(8;21)	1,80 *	1,02	0,92	1,10	0,97	1,08	0,47 **	0,84	1,39
AML t(11q23)/MLL	2,38 ***	0,82	1,16	1,06	0,85	1,18	0,55 **	0,69	1,44 *
AML complex	3,07 ***	2,04 **	0,88	1,07	1,25	1,26	0,56 **	0,90	1,56 *
HSCs	1,00	1,00	1,00	1,00	1,00	1,00	1,00	1,00	1,00

Fold change
≤1 1.5 ≥2

* p<0.05
** p<0.01
*** p<0.001

C.

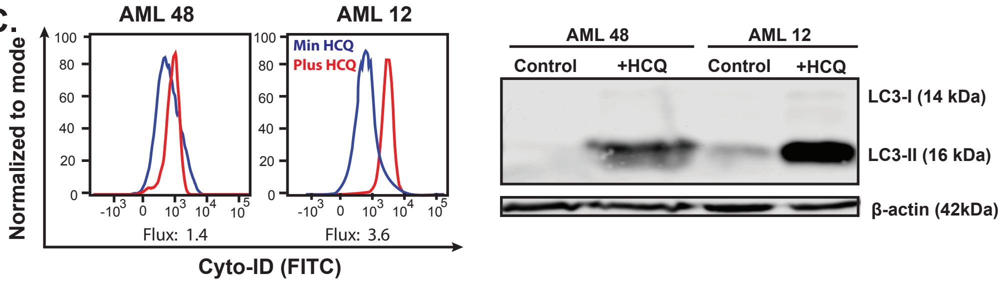

D.

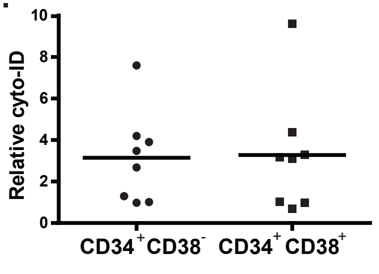

E.

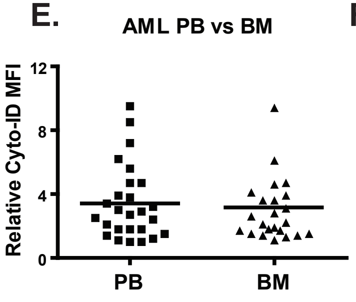

F.

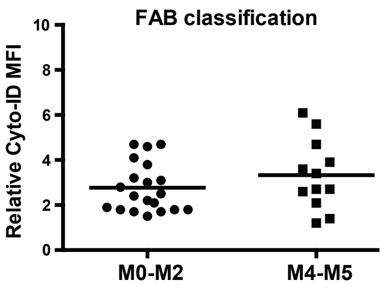

Supplementary Figure 4.

A.

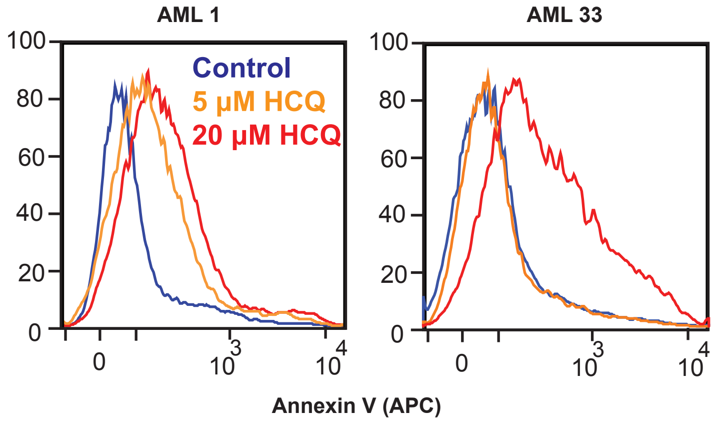

B.

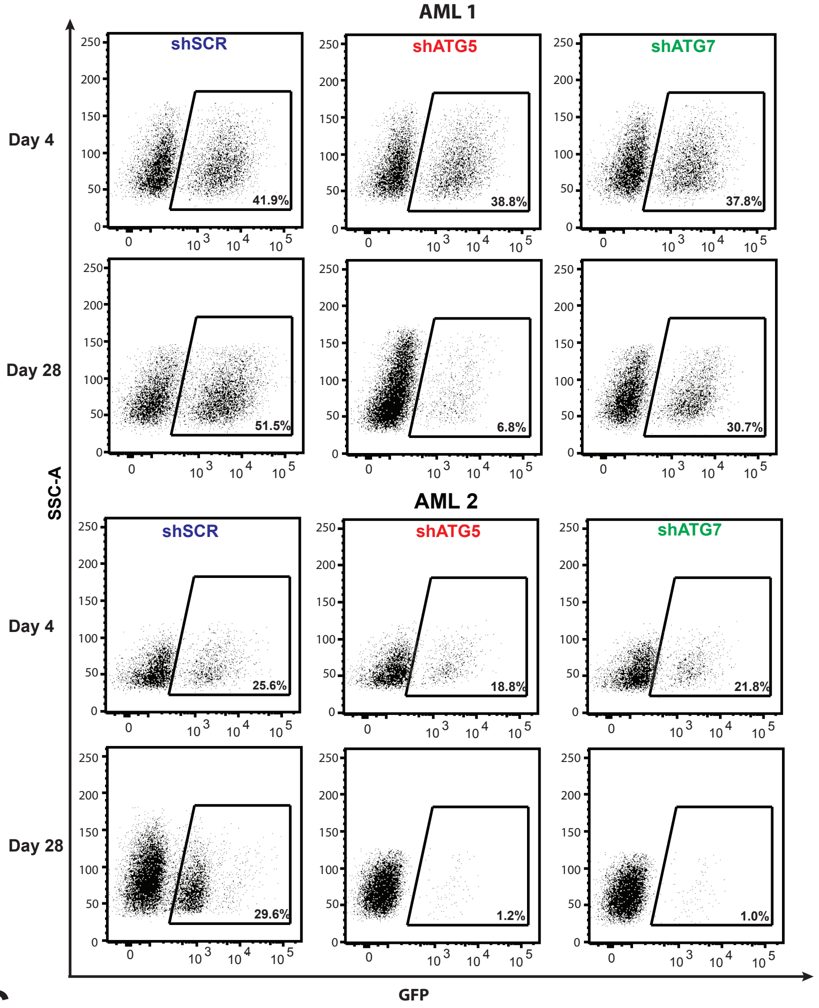

C.

	Knockdown efficiency for shATG5 and shATG7		
	shSCR	shATG5	shATG7
AML1	1,0	0,21 **	0,67 *
AML2	1,0	0,35 **	0,49 *
AML4	1,0	0,04 *	0,43 **
AML5	1,0	0,35 ***	0,50 **
AML6	1,0	0,56	0,41 *

Supplementary Figure 5.

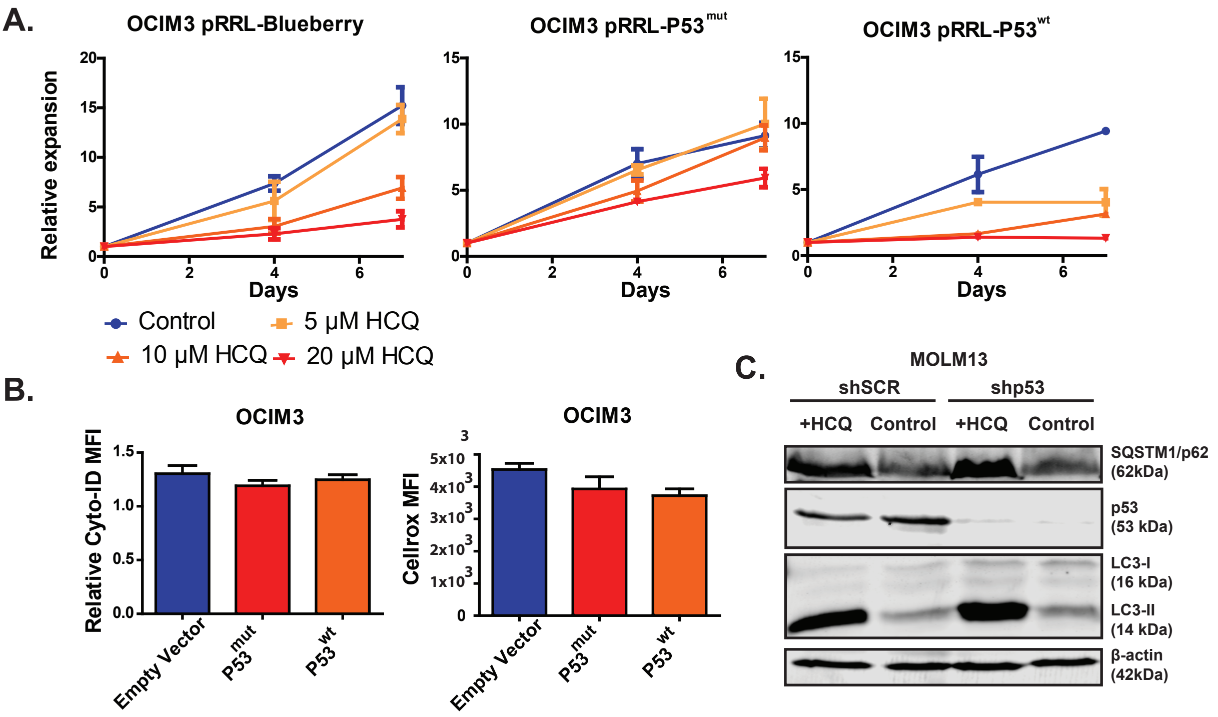

Supplementary Figure 6.

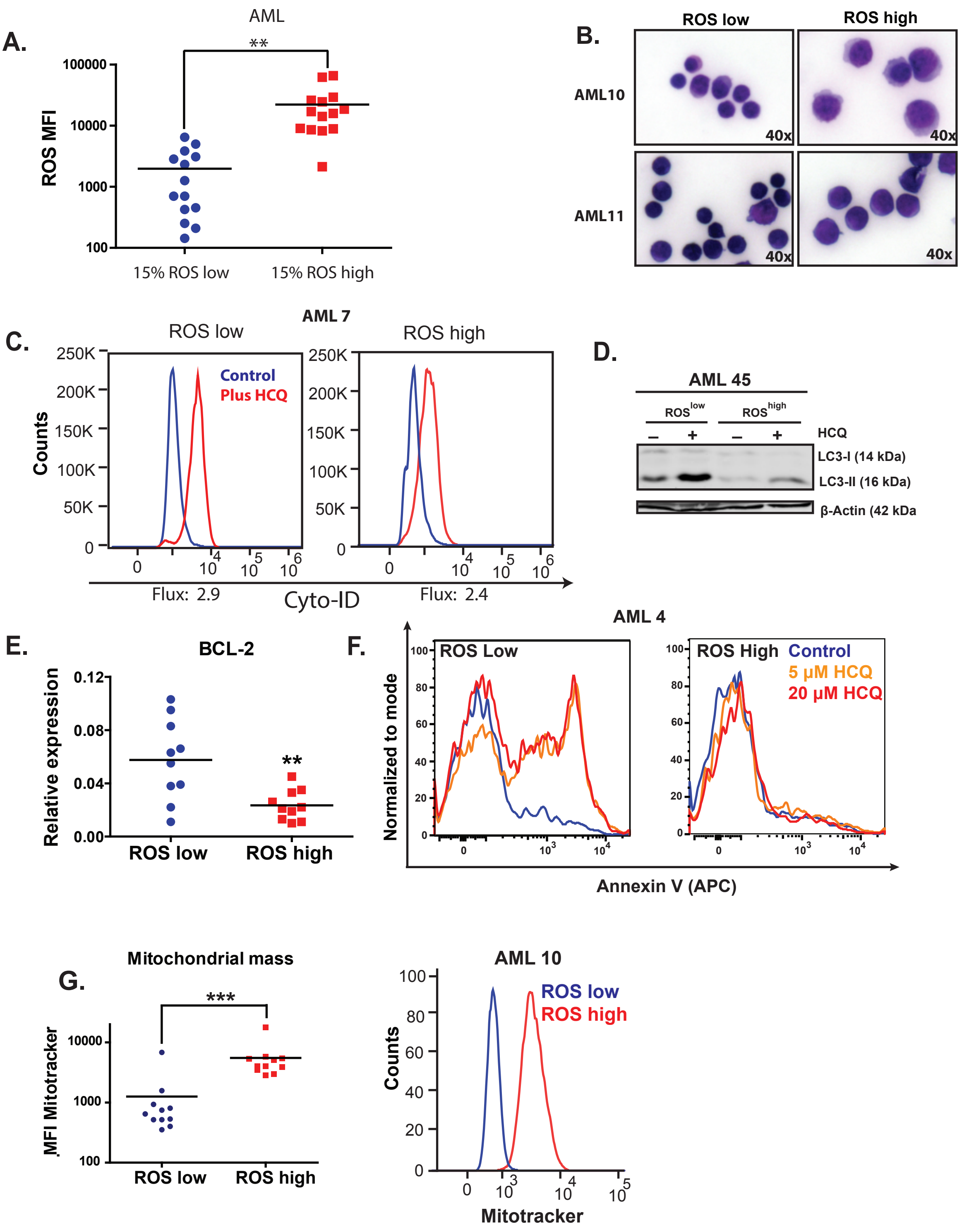

Supplementary Figure 7.

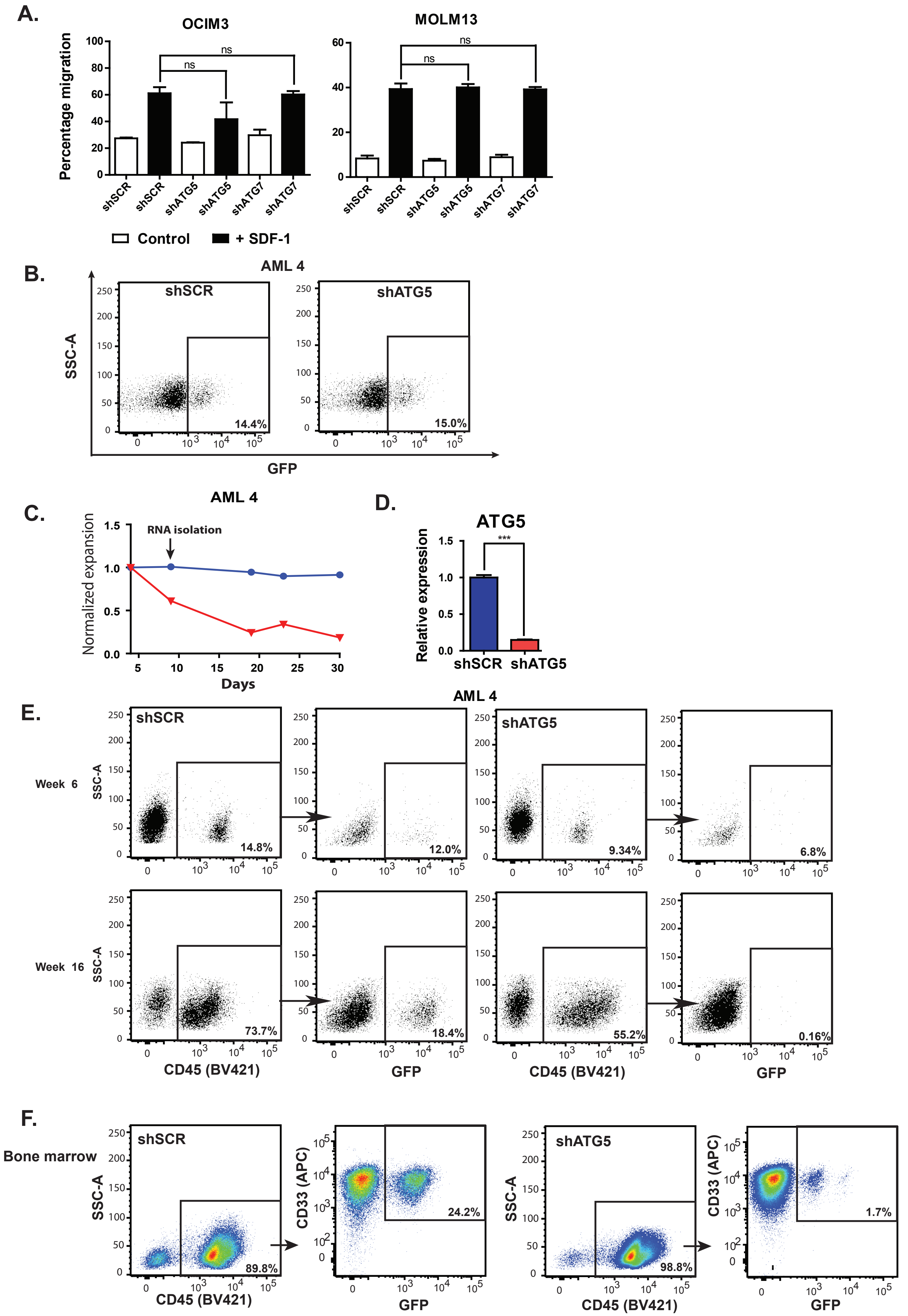

Supplementary information

Supplementary figures

Supplementary figure 1. Variation in autophagy flux between different leukemic cell lines.

A) Leukemic cell lines, expressing LC3-GFP, were treated overnight with or without BAF. LC3-pucta were analysed by fluorescent microscopy. **B)** Left panels: representative pictures of LC3-GFP expressing cells treated with or without BAF and right panels: quantification of LC3-pucta by ImageJ software. **C)** Quantification of LC3-II in HL60, OCIM3, MOLM13 and NB4 cells after HCQ treatment (n=4). **D)** Western blots of LC3-II accumulation after overnight HCQ treatment (20 µM) in leukemic cell lines, β-actin was used as loading control. **E)** Western blot showing LC3-II accumulation in HL60 and NB4 cells. HL60 and NB4 cells were pre-treated for 4 hours with PI3K inhibitor (3-MA; 3-Methyladenine) or ULK1 inhibitor (SB; SBI-0206965) before addition of HCQ. Histone H3 was used as loading control. **F**) Quantification of LC3-II levels corrected for Histone H3 levels. Error bars represent SD; *, or ** represents $p<.05$ or $p<.01$ respectively.

Supplementary figure 2. Correlation between autophagy flux and sensitivity for inhibition of autophagy in leukemic cells.

A) Left panel: table showing relative expression of ATG5, ATG7 or p53 as determined by q-RT-PCR in shSCR, shATG5, shATG7 and shP53 transduced cell lines or right panel: p53 protein level after lentiviral transduction with shSCR or shP53 in NB4 cells. **B)** Left panel: Representative pictures of LC3 puncta in OCIM3 cells transduced with shSCR, shATG5 or shATG7 at day 5 after transduction, treated overnight with or without Bafilomycin-1A (40x magnification). Right panels: Quantification of LC3 puncta in Molm13 and OCIM3, transduced with shSCR, shATG5 or shATG7. **C)** Relative expansion of leukemia cell lines cells and CB $CD34^+$ cells cultured for 10 days in the presence of different concentrations of HCQ. **D)** Annexin V levels in Molm13 and NB4 cells at day 5 after knockdown of ATG5 or ATG7. **E)** Fold increased expression measured by q-RT-PCR, of P53, BAX, PUMA and PHLDA3 after 4 days 20 µM HCQ treatment in MOLM13 and NB4 compared to untreated control. **F)** Percentage of Annexin V positive cells in shSCR or shp53 transduced NB4 cells, at day 4 after treatment with different concentrations of HCQ. **G)** q-RT-PCR for BAX and PUMA in shSCR and shP53 transduced NB4 cells, treated with 20 µM HCQ for 4 days. **H**) Relative cell expansion of OCIM3 cells double transduced with shp53-GFP or shSCR-GFP in combination with shSCR-mCherry or shATG5-mCherry. Error bars represent SD; *, ** or *** represents p<.05, p<0.01 or p<.001 respectively.

Supplementary figure 3. Variation in autophagy levels between different AMLs, independent of the differentiation status

A) Normalized gene expression (measured by q-RT-PCR) of ATG5 and ATG7 in AML $CD34^+$ cells compared to NBM $CD34^+$ cells. **B**) Heat-map showing fold-change gene expression of autophagy genes in different subtypes of AMLs, relative to expression in normal HSCs. All displayed expression data was acquired from the publicly available expression database; Bloodspot.[30]. **C)** Left panels: relative Cyto-ID values or right panels: LC3-II accumulation on Western blot of two AML $CD34^+$ samples treated overnight with or without 20 µM HCQ **D**) Relative Cyto-ID measurements in sorted $CD34^+CD38^-$ and $CD34^+CD38^+$ AML blasts after 3 days of culturing on MS5 stroma. **E**) Autophagy flux, measured by relative Cyto-ID measurements in AML $CD34^+$ cells derived from bone marrow (BM) or peripheral blood (PB). **F)** Autophagy flux in AML cells with myeloid (M0-M2) or monocytic background (M4-5). Error bars represent SD; *, ** or *** represents p<.05, p<0.01 or p<.001 respectively.

Supplementary figure 4. Inhibition of autophagy triggers apoptosis in primary AML $CD34^+$ cells

A) Representative FACS plot showing Annexin V levels in AML $CD34^+$ cells treated with 5 or 20 µM HCQ for 72 hours. **B)** Representative FACS plots showing the % GFP positive cells at day 4 and day 28, for AMLs transduced with shSCR, shATG5 or shATG7-GFP. **C)** Table showing relative expression of ATG5

or ATG7 as determined by q-RT-PCR in shSCR, shATG5 or shATG7 transduced AMLs. RNA isolated at day 7 from FACS-sorted shSCR-GFP, shATG5-GFP or shATG7-GFP positive cells. Error bars represent SD; *, ** or *** represents p<.05, p<0.01 or p<.001 respectively.

Supplementary figure 5. Inhibition of autophagy triggers a p53 dependent apoptosis response

A) Expansion curves of OCIM3 cells transduced with pRRL-mBlueberry, pRRL-p53-mBlueberry or pRRL-p53^{R273H}-mBlueberry cultured in the presence of different concentrations of HCQ, n=2. **B)** OCIM3 cells transduced with pRRL-mBlueberry, pRRL-p53-mBlueberry or pRRL-p53^{R273H}-mBlueberry, cultured for 3 days. Autophagy flux and ROS levels were subsequently determined by Cyto-ID and CellROX respectively. **C**) Western blot showing LC3-II or p62 accumulation and p53 protein levels in MOLM13 cells transduced with shSCR or shP53 treated overnight with or without 20 µM HCQ.

Supplementary figure 6. Autophagy is higher in the ROS low population of AML blasts

A) CellROX MFI in a panel of AML CD34^{+} cells (N=14) and CB CD34^{+} (N=6). **B)** Representative microscopy pictures of sorted AML CD34^{+} (n=2) ROS^{low} and

ROS^{high} cells stained with May-Grünwald *(*MGG*)* (40x magnification). **C)** Representative Cyto-ID stainings in ROS^{low} vs ROS^{high} cells treated with or without HCQ. **D)** Facs-sorted ROS^{low} or ROS^{high} cells of two CD34^{+} enriched AMLs were treated overnight with HCQ and LC3-II accumulation was detected by Western blotting. β-Actin was used as loading control. **E)** Validation of BCL-2 expression in FACS sorted ROS^{low} or ROS^{high} cells, determined by q-RT-PCR. **F)** Representative FACS plot showing Annexin V levels in ROS^{low} and ROS^{high} AML CD34^{+} cells treated with different concentrations of HCQ. **G)** Left panel: mitochondrial mass within ROS^{low} and ROS^{high} AML CD34^{+} cell fractions. **Right panel:** representative FACS plots showing MitoTracker MFI in AML ROS^{low} and ROS^{high} cells. Error bars represent SD, ** or *** represents $p<0.01$ or $p<.001$ respectively.

Supplementary figure 7. Knockdown of ATG5 in AML CD34^{+} blasts results in impaired engraftment

A) Migration towards SDF-1 of shSCR, shATG5 or shATG7 transduced OCIM3 or MOLM13 cells, 3 days after transduction, evaluated in a transwell assay. **B)** GFP percentage of transduced AML CD34^{+} cells at the day of injection. **C)** *In vitro* expansion curve of shSCR or shATG5 transduced AML CD34^{+} cells. **D)** Validation of ATG5 knockdown by q-RT-PCR of *in vitro* expanded GFP sorted AML CD34^{+}cells. **E)** Representative FACS plots from one individual mouse within each group, showing huCD45 and GFP percentages (week 6 and 16). **F)**

Representative FACS plots showing huCD45 percentages in mice bone marrow and the GFP percentages and CD33 percentage within huCD45^{+} cells

Supplementary material and methods

Antibodies and reagents

The following anti-human antibodies were used: mouse anti-SQSTM1/p62 (sc-28359) and rabbit (sc-130656) or mouse (sc-47778) anti-Actin, mouse anti-p53 (sc-126) from Santa Cruz (Santa Cruz, CA, USA), Mouse anti-LC3 (5F10, 0231-100) from Nanotools (Munich, Germany) and Mouse anti-Histone H3 was obtained from Abcam (Cambridge, UK), Hydroxychloroquine (HCQ), Bafilomycin-A1 (BafA1), Nutlin-3A, 3-Methyladenine (3-MA) and SBI-0206965 (SB) were obtained from Sigma-Aldrich.

Western blotting

Western blot analysis was performed using standard techniques. In brief, cells were lysed in Laemmli buffer and boiled for 5 min. Equal amounts of total lysate were analyzed by SDS-polyacrilamide gel electrophoresis. Proteins were transferred to polyvinylidene difluoride (PVDF) membrane (Millipore, Billerica, Massachusetts, USA) by semidry electroblotting. Membranes were blocked in Odyssey blocking buffer (Westburg, Leusden, the Netherlands) prior to

incubation with the appropriate antibodies according to the manufacturer's conditions. Membranes were washed, incubated with secondary antibodies labeled with alexa680 or IRDye800 (Invitrogen, Breda, the Netherlands) and developed by Oddysee infrared scanner (Li-Cor Biosciences, Lincoln, USA).

Fluorescence and light microscopy

pRRL-LC3-GFP or pRRL-GFP transduced MOLM13 or OCIM3 cell lines were treated for 16 hrs with or without Bafilomycin. Cytospins of cells were stained with DAPI (Nuclear stain), washed with PBS and subsequently analyzed by fluorescent microscopy (Leica DM6000B, Amsterdam, The Netherlands) GFP-puncta, as a measure for autophagy, were quantified using Image J software. Cytospins of sorted ROS low and high cells AML cells were stained with May-Grünwald-Giemsa (MGG) staining and analyzed by microscope (Leica DM3000).

***In vivo* transplantations into NSG.** Before *in vivo* transplantation, mice were sublethally irradiated (1.0 Gy). Following irradiation, mice received filter sterilized neomycin (3.5g/L) via drinking water for 2 weeks. The mice were injected IV (Lateral tail vein) with 0.5 or 1.0 x 10^6 unsorted $CD34^+$ cells for AML4 and AML5 respectively, which were transduced with shSCR-GFP, or shATG5-GFP. Four to five mice were injected for each group. The transduction levels were >14% or >32% GFP for AML4 and AML5 respectively, at the time of injection. Human cell

engraftment was analyzed in peripheral blood (PB) by FACS analysis with intervals of at least 2 weeks, starting from week 6. PB was collected via submandibular puncture.

Migration assay

Migration assay of shSCR, shATG5 or shATG7 transduced OCIM3 and MOLM13 cells was performed in a transwell system (Corning Costar, Cambridge, UK) with an 8 μm pore size. Three days after transduction, cells were resuspended in 100 μL medium and added to the upper chamber; 600 uL of medium with and without 100 ng/mL stromal derived factor-1 was added to the lower chamber. Cells were incubated for 4 hours at 37°C and migrated and non-migrated cells were counted by flowcytometry using counting beads.

Supplementary Tables

**Table S1** leukemic cell lines	
HL60 (p53 null)	MOLM13 (p53 wt)
THP1 (p53 mut)	NB4 (p53 mut)
K562 (p53 mut)	OCIM3 (p53 wt)
KG1A (p53 mut)	

Supplementary table 1: list of cell lines with the p53 status. wt, wild type; mut, mutated.

Table S2 Patient characteristics

#	Age	Male/ Female	PB/ BM	$CD34^+$ %	Risk	Demonstrated mutations	karyotype
1	69	M	PB	74	Adv	TP53	t(3;5),-5
2	49	F	BM	84	Fav	IDH1	Inv16
3	69	F	PB	84	Int	IDH2, FLT3-ITD	NK
4	60	M	PB/ BM	39	Adv	FLT3-ITD	t(3;5)(q23;q33),+8
5	48	F	BM	30	Fav		Inv(16)
6	52	M	PB	30	Int	DNMT3A, IDH2, FLT3-ITD	NK
7	42	F	PB	42	Adv	FLT3-ITD	Complex karyotype
8	61	F	PB	61	Adv	FLT3-ITD	t(11;20)(p15;q11.2)
9	52	M	PB	52	Int	FLT3-ITD	NK
10	74	F	PB	35	Adv	TP53	Complex karyotype
11	66	F	BM	8	Int	FLT3-ITD	NK
12	71	M	BM	11	Adv	TP53	Complex karyotype
13	68	M	PB	86	Int	IDH2	+11

Supplementary table 2: Abbreviations: M, male; F, female; PB, peripheral blood; BM, Bone marrow; Fav, favorable; Int, intermediate; Adv, adverse; TP53, Tumor protein P53; IDH1, isocitrate dehydrogenase 1; IDH2, Isocitrate dehydrogenase 2; FLT3-ITD, fms-like tyrosine kinase 3 internal tandem

duplication; DNMT3A, DNA Cytosine-5-Methyltransferase 3 Alpha; NK, normal karyotype.

**Table S3** Patient characteristics						
**AML Risk-group**	***n***	**(%) M**	**($\tilde{x}$)Age (range)**	**(%) FLT3-ITD**	**(%) NPM mutation**	**(%) TP53 mutation**
Favorable	6	33	48 (28-74)	0	0	0
Intermediate	28	42	54 (17-78)	43	25	0
Adverse	17	47	62 (23-74)	29	0	35

Supplementary table 3: Abbreviations: *n*, number of samples; $\tilde{x}$, median; M, male; FLT3-ITD, fms-like tyrosine kinase 3 internal tandem duplication; NPM, nucleophosmin-1; TP53, Tumor protein P53.

**Table S4** Characteristics of AML patients studied for ATG5 and ATG7 expression			
**AML Risk-group**	***n***	**(%) M**	**($\tilde{x}$)Age (range)**
Favorable	2	0	59.5 (59-60)
Intermediate	19	47	59 (31-75)
Adverse	5	20	54 (42-68)

Supplementary table 4: Abbreviations: M, male; $\tilde{x}$, median.

**Table S5** FACS antibodies			
**Antibody**	**Fluorochrome**	**Clone number**	**Company**
Anti-CD19	BV785	HIB19	BioLegend
Anti-CD33	APC	WM-53	BioLegend
Anti-CD34	Pe-Cy7	8G12	BD Pharmingen
Anti-CD38	FITC	HIT2	BD Pharmingen
Anti-CD45	BV421	HI30	BioLegend

Supplementary table 5: list of antibodies used for FACS.

**Table S6** primer sequences		
**Gene**	**sequence**	
**LC3**	Fw	5′-CGCACCTTCGAACAAAGAGTAG-3′
	Rev	5′-AGCTGCTTCTCACCCTTGTATC-3′
**VMP1**	Fw	5′-CAGATGAAGAGGGCACTGAAGG-3′
	Rev	5′-CTCCGATTGCTGTACCGATACC-3′
**ATG10**	Fw	5′-GGGAATGGAGACCATCAAAG-3′
	Rev	5′-GGTAGATGCTCCTAGATGTG-3′
**BCL-2**	Fw	5′-GAGGCTGGGATGCCTTTGTG-3′
	Rev	5′-GGGCCAAACTGAGCAGAGTC-3′
**P53**	Fw	5′-GAGATGTTCCGAGAGCTGAATGAGGC-3′
	Rev	5′-TCTTGAACATGAGTTTTTTATGGCGGGAGG-3′
**ATG5**	Fw	5′-GGGCCATCAATCGGAAAC-3′
	Rev	5′-AGCCACAGGACGAAACAG-3′
**ATG7**	Fw	5′-CGTTGCCCACAGCATCATCTTC-3′
	Rev	5′-TCCCATGCCTCCTTTCTGGTTC-3′
**Beclin-1**	Fw	5′-CATGCAATGGTGGCTTTC-3′
	Rev	5′-TCTCCACATCCATCCTGTAG-3′
**BAX**	Fw	5′-CCAGCAAACTGGTGCTCAAG-3′
	Rev	5′-GGAGGCTTGAGGAGTCTCAC-3′
**PUMA**	Fw	5′-GACCTCAACGCACAGTACG-3′
	Rev	5′-GGCAGGAGTCCCATGATGAG-3′
**PHLDA3**	Fw:	5′-GGACCCTCGTGTCCTAAACC-3′
	Rev:	5′-CCTTGCCACATGGAGCACAG-3′
**p21**	Fw:	5′-CGACTGTGATGCGCTAATGG-3
	Rev:	5′-CGTTTTCGACCCTGAGAG-3′
**RPL27**	Fw	5′-TCCGGACGCAAAGCTGTCATCG-3′
	Rev	5′-TCTTGCCCATGGCAGCTGTCAC-3′
**RPS11**	Fw	5′-AAGATGGCGGACATTCAGAC-3′
	Rev	5′-AGCTTCTCCTTGCCAGTTTC-3′
**FOXO3A**	Fw	5′-ATAAGGGCGACAGCAACAG-3′
	Rev	5′-CTCTTGCCAGTTCCCTCATTC-3′
**SOD1**	Fw	5′-GTGCAGGGCATCATCAATTTCG-3′
	Rev	5′-AATCCATGCAGGCCTTCAGTC-3′
**SOD2**	Fw	5′-CCTACGTGAACAACCTGAAC-3′
	Rev	5′-AGAGCTATCTGGGCTGTAAC-3′
**Catalase**	Fw	5′-AGACTCCCATCGCAGTTC-3′
	Rev	5′-CCAACGAGATCCCAGTTACC-3′

Supplementary table 6: list of primers used for q-RT-PCR. Abbreviations: Rev, reverse; Fw, forward.
